# Homogenisation of Vegetation in Irish Semi‐Natural Grasslands

**DOI:** 10.1002/ece3.73231

**Published:** 2026-03-10

**Authors:** Oliver Lynch Milner, Astrid Wingler, Fiona Cawkwell, Karen L. Bacon

**Affiliations:** ^1^ Botany & Plant Sciences, School of Natural Science, University of Galway Galway Ireland; ^2^ School of Biological, Earth & Environmental Sciences, University College Cork Cork Ireland; ^3^ Sustainability Institute, University College Cork Cork Ireland; ^4^ Department of Geography University College Cork Cork Ireland

**Keywords:** Irish grasslands, plant diversity, resurvey, semi‐natural grasslands, vegetative homogeneity

## Abstract

Grasslands represent an important source of vegetative diversity and provide a range of important ecosystem services. Semi‐natural grasslands in Europe face a variety of threats due to changing management practices and other anthropogenic pressures. This study investigates vegetative changes in 12 semi‐natural grassland sites in Ireland over an approximately 15‐year period. Sites for three habitat types (GS1—dry calcareous & neutral grassland, GS3—dry‐humid acid grassland and GS4—wet grassland) were selected from the 2007–2012 Irish Semi‐natural Grassland Survey and resurveyed in 2023. The resurveyed sites showed a minor shift in vegetative composition in terms of species richness, but non‐metric multidimensional scaling suggests that the grasslands are increasingly homogenous with habitat types having become less distinct. While both species losses and gains were observed, almost half of the forb species decreased in frequency, and some of the rarer species were lost. This raises concerns about the mid‐ and long‐term diversity of Irish semi‐natural grasslands and suggests that careful management aimed at protecting diversity is required.

## Graphical abstract

A re‐survey of 12 Irish grassland sites revealed a decrease in species number and an apparent shift towards homogeneity of species diversity over the last 12–15 years. This raises concerns about management and species diversity/conservation.

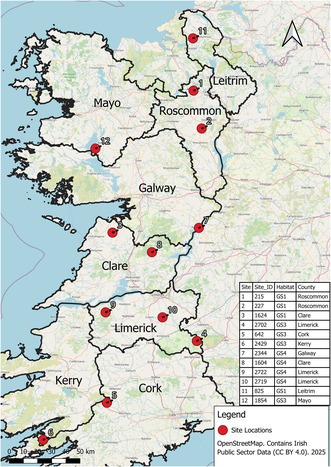

## 1 Introduction

Twenty to thirty per cent of the terrestrial surface of the Earth is covered by grassland (White et al. 2000; Ramankutty et al. 2008). Grasslands are ecosystems dominated by grasses, with a lack of woody vegetation (Gibson 2009; Janišová et al. 2011; Dengler et al. 2014; Bardgett et al. 2021) and are a mosaic of graminoid (grasses, sedges and rushes) and broad‐leaved forb species (Stace 2019). Grasslands range from natural and semi‐natural environments to intensively managed agricultural grasslands. Natural grasslands are those with no anthropogenic management, where the succession of woody vegetation is prevented by other factors, such as climate (Bardgett et al. 2021). Contrastingly, intensively managed grasslands have undergone substantial alterations for agricultural production and are maintained by intensive grazing or mowing and high chemical inputs, such as fertilisers and pesticides (Bilotta et al. 2007; Grange et al. 2021). Semi‐natural grasslands, the focus of this study, are in the middle of this management gradient. They are extensively managed but with little to no chemical inputs, which results in these grasslands being species‐rich (Bullock et al. 2011; Janišová et al. 2014), particularly compared to intensively managed grasslands. Plant diversity of semi‐natural grasslands is high, often up to 81 species/m^2^ (Bastow Wilson et al. 2012; Chytrý et al. 2015). The inclusion of management practices such as grazing or mowing in the absence of nutrient addition, reduces the aboveground biomass, suppresses the dominance of grass and other competitive species, and leads to reduced competition for both light and niche space (Borer et al. 2014; Pulungan et al. 2019) ultimately increasing plant species diversity. When management ceases, abandonment occurs (O'Neill et al. 2013; Valkó et al. 2018). The resulting increase in biomass (Borer et al. 2014; Valkó et al. 2018; Eskelinen et al. 2022; McKeon et al. 2022) leads to increased plant litter accumulation (Kelemen et al. 2014; McKeon et al. 2022) and the competitive exclusion of plant species richness, in particular forbs, due to reduced light availability (Borer et al. 2014; Valkó et al. 2018; Eskelinen et al. 2022; McKeon et al. 2022).

In addition to abandonment, intensification by adding nutrients to typically nutrient poor semi‐natural grasslands, results in a reduction of species diversity (Hautier et al. 2009; Bonanomi et al. 2013). This is due to increased dominance of grasses and other competitive species (Cousins and Eriksson 2008; Bonanomi et al. 2013; Ceulemans et al. 2013).

Since the 1950s, Europe has experienced declines of between 47% and 97% in the area of semi‐natural grasslands (Fuller 1987; Ridding et al. 2015; Aune et al. 2018), as well as changes to the species diversity and composition (e.g., Duprè et al. 2010; Newton et al. 2012; Pipenbaher et al. 2013; Diekmann et al. 2014, 2019). Some studies reported declines in species diversity (e.g., Ridding et al. 2021), while others reported increases in diversity (e.g., Mitchell et al. 2017) or no overall change (e.g., Diekmann et al. 2014). The most consistent changes in grasslands reported were changes in species composition (e.g., Pykala 2003; Duprè et al. 2010; Diekmann et al. 2014, 2019; Giarrizzo et al. 2017; Watts et al. 2019; Buzhdygan et al. 2020). Examples include increases in taller more competitive species, as well as graminoid species, at the expense of forb and herb species, and the loss of specialist grassland species (Berlin et al. 2000; Diekmann et al. 2014; Bauer and Albrecht 2020). While changes in diversity may not be inherently obvious, changes in composition can indicate degradation of the grassland habitat, mainly through the process of homogenisation. This degradation and compositional change has been attributed to the threats described above, most significantly agricultural intensification and land‐use change (Cousins and Eriksson 2008; Johansson et al. 2008; Ridding et al. 2015; Aune et al. 2018; Prangel et al. 2023).

While grasslands contribute to almost 62% of Ireland's land cover, most of them are intensively managed, and only 0.7% of the total land area, or 1% of the agricultural area, is semi‐natural grassland (CSO 2023). However, similar to other European countries, agricultural intensification and land‐use change threaten Irish semi‐natural grasslands. For example, afforestation of these grasslands, especially wet grasslands, has resulted in declines in area (Buscardo et al. 2008). It is estimated that approximately 46,200 ha of grassland were converted to woodland between 2000 and 2018 (CSO 2023). In addition, over 70% of Ireland's semi‐natural grasslands that are protected under the EU Habitat Directive Annex I are reported as being negatively impacted by agricultural activities (NPWS 2019).

Between 2007 and 2013, the Irish Semi‐natural Grassland Survey (ISGS) surveyed 1192 grassland sites across Ireland, collecting a total of 4544 grassland relevés (O'Neill et al. 2013). This was the first nationwide survey of semi‐natural grasslands in Ireland, collating data on species composition, habitat classification and conservation status of both Annex I and non‐Annex I grasslands. This survey has provided invaluable baseline information for future studies, in particular for Annex I grassland assessments. However, as Annex I grassland only accounts for 12.4% of the total area of grassland surveyed by the ISGS, grasslands that have not been afforded this status have not been re‐surveyed. This leaves a considerable research gap on how the diversity and species composition, and subsequently conservation status, has changed since the ISGS in these grasslands.

The aim of this study is to address this research gap and determine changes in plant biodiversity and species composition of non‐Annex I semi‐natural grassland habitats. To achieve this, 12 sites were selected from the original ISGS and resurveyed to determine how vegetation may have changed in the intervening years. Even though the selected sites are still classed as semi‐natural grassland habitats, we hypothesise that changes in plant species composition will show signs of degradation, indicating homogenisation of the habitats.

## 2 Methodology

### 2.1 Site Selection

**FIGURE 1:**
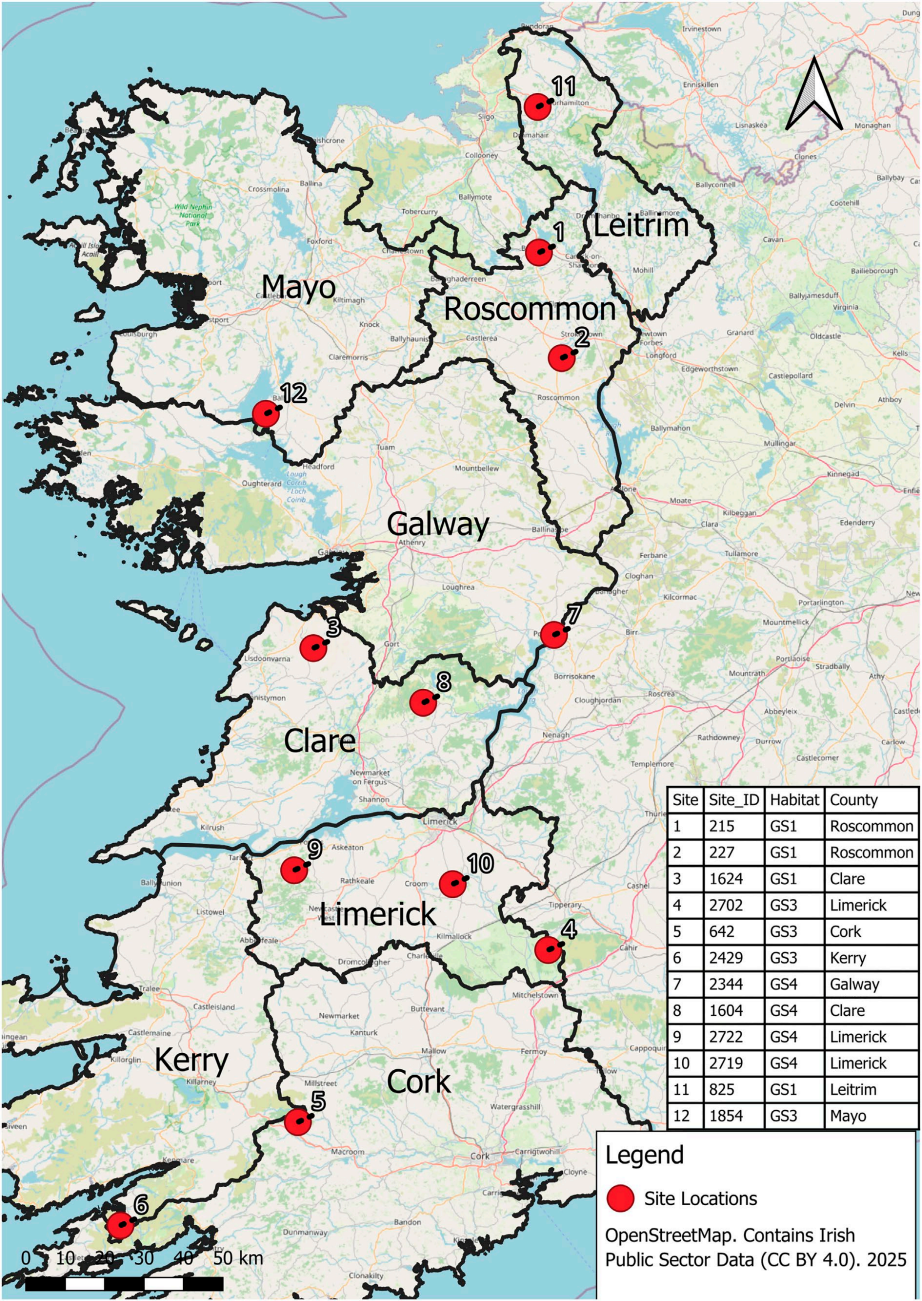
Location and distribution of the 12 sites in the study (GS1 = 4 sites, GS3 = 4 sites, GS4 = 4 sites). ISGS site identification and Fossitt habitat classification for each site are included on the figure.

The 12 sites (Figure 1) were selected from the ISGS dataset, which is publicly available as a GIS resource, and as an Access database (O'Neill et al. 2013). These 12 sites encompass three habitat types, with four sites selected per habitat, namely GS1—Dry calcareous & neutral grassland, GS3—Dry‐humid acid grassland and GS4—Wet grassland (Fossitt 2000). These were the three most frequently recorded habitats in the ISGS, with the remaining two Fossitt grasslands (GS2 and GM1) having low coverage in the ISGS and not included in the resurvey.

Selection criteria for sites were as follows: (1) located in the eight counties listed above; (2) having an area greater than nine hectares; (3) having a semi‐natural grassland habitat cover of > 55% of the target habitat type in the ISGS; and (4) having a continuous area as opposed to being composed of separate grassland parcels. Criterion 1 was necessary due to the prevalence of semi‐natural grasslands in the West of Ireland and to keep differences in climate and management between sites as minor as possible. Criteria 2 and 4 were specified to support analysis via remote sensing that was a key component of the wider study but are not discussed further here. Criterion 3 ensured that the majority of the site contained the desired habitat type. The ISGS diversity and the presence of Annex I habitats were not considered at the site selection stage to ensure a variety of diversities were included. After the above criteria were applied to the list of ISGS sites, four sites of each habitat were randomly selected. Landowner permission was obtained to survey all sites. If a monument was present on the site and/or the site was located in a Special Area of Conservation relevant legislation was followed, for example, Section 5 (8) of the 1987 Act (Register of Historic Monuments), the National Monuments (Amendment) Act 1994 and the (European Communities (Birds and Natural Habitats) Regulations 2011). None of the selected sites were classed as Annex I grasslands.

#### 2.1.1 Site and Relevé Survey

Fieldwork was conducted between the June 12 and the August 8, 2023. The field survey methodology followed that of the ISGS.

As with the ISGS, relevés of 4 m^2^ (2 m × 2 m quadrats) were surveyed to describe the current vegetation composition and diversity. Where possible, the StableGrass relevés were repeats of the ISGS relevés but additional relevés were collected where the ISGS relevé was no longer accessible, had dangerous livestock, or no longer contained semi‐natural grassland. A total of 54 relevés were surveyed across the 12 sites, with 44 of these being the same location as in the ISGS survey.

For each relevé, vascular plants and bryophyte species were identified, following the nomenclatures of Stace (2019) and Atherton (2010), respectively. In most cases, species level identifications were obtained except for particularly difficult taxa, for example, *Hieracium* spp, or species that are difficult or impossible to identify vegetatively without flowers, for example, *Viola riviniana*/*reichenbachiana* or non‐flowering *Carex* spp. Bryophytes were identified in the field where possible, with unidentified specimens being vouchered and identified in the laboratory. For all species, a percentage cover scale, as recommended by O'Neill et al. (2013), was applied (0.1%, 0.3%, 0.5%, 0.7%, 1%, 3%, 5% and 7%; 10%; and subsequently 5% increments). In each relevé the following environmental variables were collected: graminoid cover (%), forb cover (%), forb:graminoid ratio, median sward height (calculated from three random areas in relevé) and percentage cover of the following: bare rock, bryophyte layer, litter, bracken, scrub. The vegetation/habitat type of each relevé was recorded using Fossitt (2000) and raw data are available in the Supporting Information.

#### 2.1.2 Data Analysis

##### 2.1.2.1 Overview

Two datasets were analysed: (1) ISGS relevé data for the 12 sites (*n* = 51 relevés) and (2) StableGrass relevé data (*n* = 54 relevés). Paired analysis was possible for 44 relevés that were the same in both surveys. As the ISGS dataset recorded species cover in the DOMIN scale, conversion to percentage cover was required to allow for quantitative analysis (O'Neill et al. 2013). All analyses were conducted in the R Statistical Environment (R Core Team 2024).

##### 2.1.2.2 Changes in Species Diversity

Species Richness, Gini‐Simpson's Diversity (hereafter referred to as ‘Simpson's Diversity’), and Simpson's Evenness were calculated for all relevés using R functions ‘specnumber’ (Species Richness) and the ‘diversity’ from the ‘Vegan’ package (Oksanen et al. 2024). In this case, Species Richness refers to the number of unique species present in the relevé.

A Kruskal–Wallis analysis was used to test for differences in Species Richness across the three Fossitt habitats, with ANOVA tests used for Simpson's Diversity and Simpson's Evenness. If a significant difference was revealed, pairwise analysis was conducted (pairwise Wilcoxon Rank Sum test for Species Richness, and Tukey's HSD test for Simpson's Diversity).

For the 44 paired relevés surveyed in the ISGS and StableGrass, a paired *t*‐test was used to test for differences in both Species Richness and Simpson's Diversity, and a Wilcoxon signed‐rank test was used to test for Simpson's Evenness. Differences in diversity at the Fossitt habitat level were also assessed.

##### 2.1.2.3 Changes in Sites and Habitats

Non‐metric multidimensional scaling (NMDS) was conducted using the ‘metaMDS’ function of the Vegan package (Oksanen et al. 2024). The Bray‐Curtis distance measure was used, as it is appropriate for vegetation community data (McCune and Grace 2003). NMDS plots were created for the 44 coincident ISGS and StableGrass datasets. Convex hulls were fitted to show the relevés grouping with respect to the three Fossitt habitats. The ISGS and StableGrass datasets were both aggregated to site by calculating the mean abundance of all species across the relevés to give an overall data point for each site in both the ISGS and StableGrass survey. The significance of differences in species composition on both the relevé and site‐level NMDS plots was tested using a Multi‐Response Permutation Procedure (MRPP) (McCune and Grace 2003). Homogenisation was further assessed via comparing the dispersion ‘betadisper’ and the dissimilarity ‘vegdist’ between relevés from both surveys.

##### 2.1.2.4 Species Frequency

Species frequency was calculated at both the relevé and the site level for both surveys to determine changes in frequency between the two survey periods. Relevé‐level frequency refers to the number of relevés out of the total that a species was recorded in, whereas site‐level frequency refers to the number of sites (out of 12) that the species was recorded in. The rate of change (StableGrass frequency—ISGS frequency) was calculated for all relevé and site frequencies. Paired *t*‐tests/Wilcoxon‐signed Rank tests were used to test for significant differences between the site and relevé level frequencies. A functional group approach was also taken by dividing the species lists of both surveys into grasses, forbs, legumes, woody/shrub, pteridophytes and bryophytes, allowing for an assessment of which groups were increasing or decreasing in frequency over time.

## 3 Results

### 3.1 Changes in Diversity Since ISGS

**FIGURE 2:**
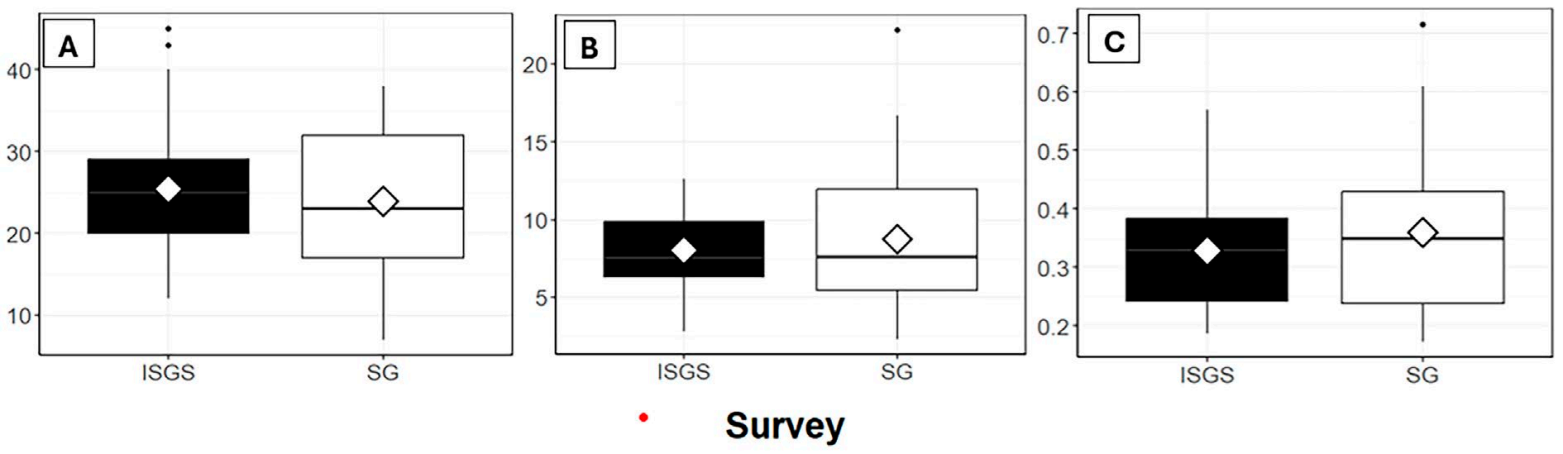
Boxplots showing the three diversity measures in the ISGS (*n* = 44 relevés) and the StableGrass (*n* = 44 relevés) survey. (A) Species Richness, (B) Simpson's Diversity and (C) Simpson's Evenness. The boxes refer to the interquartile range (top line = Q1, centre line = median, bottom line = Q3). The whiskers refer to the minimum (bottom) and maximum (top). Outliers are represented by black dots. Means are superimposed as white diamonds. Statistical tests: A: Wilcoxon signed‐rank test, *p* = 0.12; B = paired *t*‐test, *p* = 0.270; C = paired *t*‐test, *p* = 0.127.

No statistically significant differences were identified in the diversity indices between the ISGS (2007–2012) and StableGrass (2023) surveys as shown in Figure 2. As these analyses used paired statistical tests, only those relevés that were directly resurveyed from the ISGS (*n* = 44) are used and presented in the following analysis.

The mean Species Richness across the relevés was 24.8 ± 7.2 in the ISGS survey and 23.5 ± 7.8 in the StableGrass Survey (Figure 2A). However, this slight decrease was not statistically significant (Wilcoxon signed rank test, *p* = 0.116). The mean Simpson's Diversity increased slightly, but not significantly (paired *t*‐test, *p* = 0.270) from 7.87 ± 2.31 for the ISGS to 8.77 ± 4.26 for the StableGrass survey (Figure 2B). There was a slight increase in the mean Simpson's Evenness in the StableGrass survey in comparison to the ISGS, but again it was not significant (paired *t*‐test, *p* = 0.127, Figure 2C).

Despite the lack of a significant difference for all sites over time, there was a significant reduction (by 53%) in the Species Richness at Site 7 (GS4 grassland, Portumna Co. Galway) since the ISGS survey (StableGrass: mean = 13.67, and ISGS: mean = 29 species) (Shapiro–Wilk test, *p* = 0.253, paired *t*‐test, *p* = 0.02). The Simpson's Diversity index also decreased by 70% when compared to the ISGS (StableGrass: mean = 3.188, ISGS: mean = 10.543; Shapiro–Wilk test, *p* = 0.051, paired *t*‐test, *p* = 0.032). The Simpson's Evenness did not differ significantly between survey period for this site. This was the only site to have a statistically significant difference in the diversity measures between the ISGS and StableGrass surveys.

While the overall Species Richness and Simpson's Diversity and Evenness measures did not change significantly between the StableGrass and ISGS surveys, an additional analysis was conducted to determine if changes in diversity occurred at the relevé habitat level (Table 1). Relevés of the GS1 habitat had significant increases in Simpson's Diversity values since the ISGS, while GS4 relevés had significant decreases in Species Richness.

**TABLE 1:** Changes in the three diversity measures between the ISGS and StableGrass survey across the three different relevé habitats. Direction of change denoted by arrows and significance denoted by an asterisk (*).

Index	GS1	GS3	GS4
Species Richness	↑	↓	↓*
Simpson's Diversity	↑*	↑	↓
Simpson's Evenness	↑	↑	↑

*Note:* Changes are denoted by arrows: ↑ = Increase ↓ = Decrease. A significant change is denoted by an Asterisk (*). Significance: *p* < 0.05. Simpson's Diversity—GS1 = paired *t*‐test, *p* = 0.015. Species Richness—GS4 = Wilcoxon Rank‐Signed Test, *p* = 0.043. Raw data provided in Supporting Information sheet 2.

### 3.2 Changes in Sites and Habitats

**FIGURE 3:**
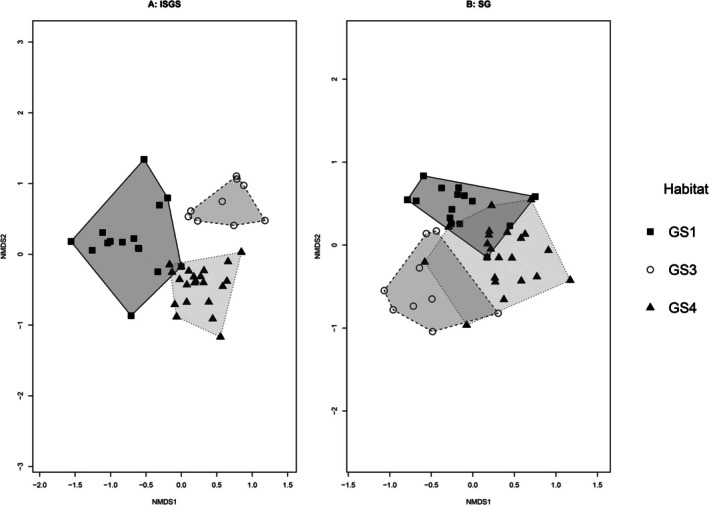
NMDS showing (A) Irish Semi‐natural Grassland Survey 2007–2012 relevés and (B) StableGrass relevés. Shape of symbols and convex hulls represent the Fossitt habitat type. Filled squares (GS1, *n* = 15), Empty Circles (GS3, *n* = 9) and Filled Triangles (GS4, *n* = 22). Only relevés that were included in both surveys formed part of this analysis. An NMDS with three dimensions (*k* = 3), was conducted as this produced the best stress values (Stress = 0.1509).

Non‐metric multidimensional scaling plots were created for both the StableGrass and ISGS vegetation datasets separately. For both surveys, the majority of relevés showed clear groupings and affinities to the Fossitt habitats (Figure 3). However, the grouping was more distinct for the ISGS, whereas for the StableGrass survey, some overlap between relevés of different habitats was observed. The shift in species composition from the ISGS to StableGrass survey was statistically significant (MRPP, *p* = 0.002), but had a very low effect size.

**FIGURE 4:**
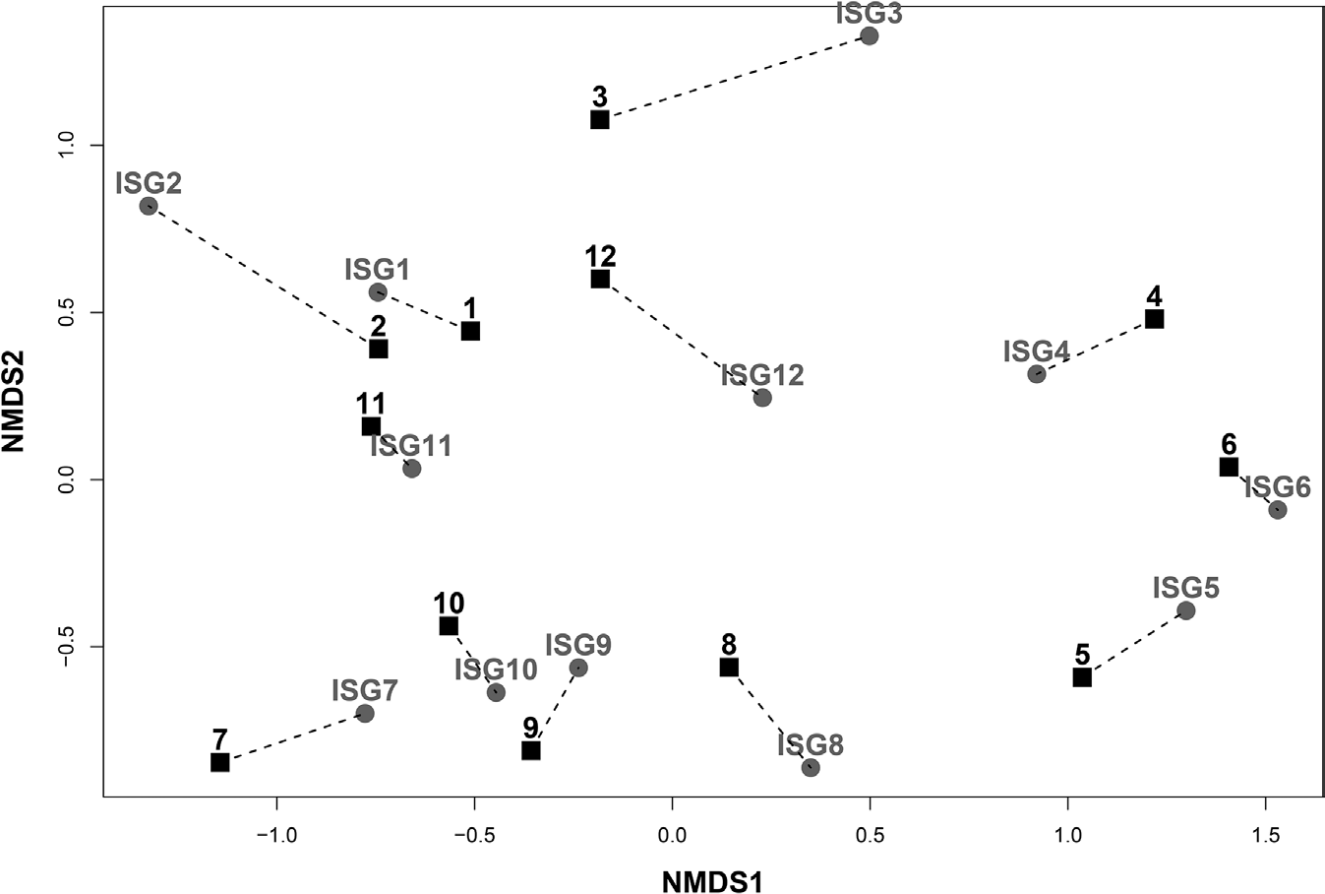
nMDS Showing the ISGS sites (grey circles, *n* = 12), and the associated StableGrass sites (black squares, *n* = 12). Each symbol represents the mean abundance for all species recorded across the relevés within the sites. Dashed lines indicate the degree of separation that occurred at each site between the two surveys.

To further test the shift in composition over time, NMDS analysis was conducted at the site level. The relevé data were aggregated to site by calculating the mean abundance of all species across the relevés within each site (using the 44 directly comparable relevés). This was done for both the ISGS and StableGrass datasets, with a data point on the NMDS plot representing each site for both the StableGrass and ISGS surveys (Figure 4). This comparison revealed a clear shift in composition for some of the selected sites since they were last surveyed in the ISGS. Sites 1, 6, 9, 10 and 11 are shown to have a stable species composition since the ISGS based on the close proximity of the two survey points. The greatest relative distances on the ordination space between the ISGS and StableGrass surveys occur for Sites 2, 3, 7 and 12, identifying notable shifts in species composition. Despite the significant MRPP (MRPP, *p* = 0.002) for the difference in composition between surveys based on relevé data (Figure 3), the same statistical test (using ‘Survey’ as a grouping variable) was not significant when used on this site‐level dataset (MRPP, *p* = 0.344).

Analysis of dispersion showed that the quadrats had not become more or less heterogeneous between surveys (*F* = 0.0778; *p* = 0.802) and a subsequent permanova showed a significant difference between paired quadrats over time, indicating a change in composition between the two survey periods (*F* = 2.459; *p* < 0.001). Overall, this suggests that the quadrats became more homogenous between the two survey periods.

### 3.3 Changes at the Species Level

Changes were assessed at the species level, including differences in species between surveys, and changes to the frequency with which each species was recorded, when compared to the ISGS. A total of 173 species were recorded for the StableGrass surveys, while 209 species were recorded for the same sites during the ISGS surveys. Across both surveys, a total of 239 species were recorded. Between the two surveys, a total of 63 species have not been re‐found in the StableGrass surveys (26%). A total of 31 species (13%) were recorded in the StableGrass survey that were not recorded in the ISGS survey. Of these 31 new species, eight were forbs, seven were graminoids, one was a pteridophyte, three were woody angiosperms and 12 were bryophytes.

Frequency was calculated at both the site and relevé level. More species decreased in frequency (111 at both the site and relevé levels) between surveys than increased or remained stable (Table 2).

**TABLE 2:** Proportion of species that decreased, increased and remained stable in frequency across the two survey periods.

Change	Site (*n* = 239 species)	Relevé (*n* = 239 species)
Decreased	46.44% (*n* = 111)	46.44% (*n* = 111)
Stable (no change)	25.52% (*n* = 61)	22.18% (*n* = 53)
Increased	28.03% (*n* = 67)	30.96% (*n* = 74)

### 3.4 Frequency Changes by Functional Group

Six different plant functional groups were described: Forbs, graminoids (grasses/sedges/rushes), leguminous, pteridophytes, woody/shrubs and bryophytes (Table 3). For this analysis, only frequency changes over relevés are included because this captures the finer scale variation in the changes of frequency in the species.

**TABLE 3:** Changes in frequency between the StableGrass and ISGS survey according to functional group.

Group	No. of species	Decreased in frequency	No change	Increased in frequency
Forbs	92	45 (49%)	21 (23%)	26 (28%)
Graminoids	61	20 (33%)	20 (33%)	21 (34%)
Legumes	6	2 (33%)	1 (17%)	3 (50%)
Pteridophytes	6	5 (83%)	0 (0%)	1 (17%)
Woody/shrubs	9	4 (44%)	1 (11%)	4 (44%)
Bryophytes	65	35 (54%)	10 (15%)	20 (31%)

The forbs, bryophytes and graminoid groups contained the highest number of species (38%, 27% and 26%, respectively). The greatest percentage decrease in frequency was observed in the pteridophyte group (83%), followed by the bryophyte group (54%) (Table 3). Despite the high proportion of species decreasing in frequency, 31% of bryophyte species were encountered in higher frequencies. Of the forb species, 49% experienced decreases in frequency across relevés (Table 3). Twenty‐three per cent of the forb species remained unchanged in frequency between the two surveys, while 28% of forbs had increased in frequency. Graminoids had similar numbers of species that decreased in frequency (33%), had no change in frequency (33%), and increased in frequency (34%). The woody/shrubs group had a 44% increase in the numbers of species that had an increased frequency.

## 4 Discussion

No significant difference in the three species diversity measures was identified between the two survey periods for the grassland sites in this study (Figures 2 and 4) on Irish semi‐natural grasslands, in contrast to findings of previous studies in other countries (e.g., Duprè et al. 2010; Newton et al. 2012; Hülber et al. 2017; Mitchell et al. 2017; Bauer and Albrecht 2020; Ridding et al. 2020; Klinkovská et al. 2024). However, a strong indication that the process of homogenisation is occurring in Irish grasslands has become apparent in this study.

Changes in species composition were identified through ordination (Figures 3 and 4), analysis of frequencies and changes at the species level (Tables 2 and 3). Homogenisation occurs when vegetation communities become more alike over time, losing the finer‐scaled variation in the vegetation. Homogenisation is a threat globally to grassland biodiversity, with moderate increases in land‐use intensity, as well as abandonment resulting in homogenisation across microbial, animal and plant taxa (Gossner et al. 2016; Schrama et al. 2023). Heterogeneity, which is an important component for promoting a high species diversity (Adler et al. 2001; Rook et al. 2004; Marion et al. 2010; Reisch and Hartig 2021), is reduced when homogenisation occurs. The increased overlap of habitats and relevés in Figure 3 evidences the view of increasing homogenisation in the semi‐natural grassland sites. An increasing trend towards homogenisation is consistent with other studies into the changing composition of grasslands (e.g., Köhler et al. 2005; Bennie et al. 2006; Halada et al. 2008; Bauer et al. 2009; Mitchell et al. 2017; Diekmann et al. 2019; Ridding et al. 2020; Pakeman and Fielding 2021; Vidaller et al. 2022).

Other processes may have also occurred either individually or in combination with homogenisation to give the resulting change in species composition. Species turnover may have also been responsible for the presence of different species but result in no detectable changes in species diversity. For example, while species may have been lost in a site, these may be replaced by other species. This scenario would have no observable difference in species richness while having a changed species composition. Some turnover is supported by our findings, although for all sites combined the number of lost species (63) was higher than the number of new species (31).

While it was expected that Species Richness, among the other diversity measures, would have decreased or changed over time, this is not always the case as seen in other studies (Berlin et al. 2000; Mitchell et al. 2017; Diekmann et al. 2019). For example, Diekmann et al. (2014) observed slight positive to no change in the Species Richness of calcareous grassland plots that were assessed over the 70‐year study period. Other studies into temporal changes in grassland vegetation reported increases in Species Richness (e.g., Newton et al. 2012; Phoenix et al. 2012; Mitchell et al. 2017). Despite this lack of an overall change, there are notable losses of diversity at the site level, with two sites undergoing considerable changes in diversity since the ISGS. Site 7 (ISGS ID 2344), a GS4 site located in Portumna Co. Galway, had a 50% reduction in the mean species richness since the ISGS. For this site, there is evidence that the management changed considerably between surveys, moving from a grazed pasture to an abandoned pasture. Other sites have evidence for an increase in grazing intensity (e.g., sites 4, 5 and 6) but not as major a change as the shift from managed with grazing to abandoned observed at site 7. This makes site 7 the only one to have a management‐driven shift in species richness. However, these changes were not statistically significant, likely due to a small sample size to compare the diversity of Site 7 between the StableGrass survey (*n* = 3 relevés) and ISGS survey (*n* = 3 relevés). Site 3 (ISGS ID 1624, GS1, Burren, County Clare) also had similar decreases in mean Species Richness but between survey differences could not be statistically tested because only one relevé was taken in the ISGS.

Many different factors could have influenced the lack of a significant difference in the diversity measures since the ISGS. The length of time (~11–15 years) since the ISGS may not have been sufficient time for significant changes to have occurred. The studies that have reported significant changes in diversity over time typically studied longer time periods than this study, ~40–100 years (Mitchell et al. 2017; Diekmann et al. 2019; Bauer and Albrecht 2020). Measures such as Species Richness can be slow to respond to changes because the grassland plant species may have long life cycles with slow intrinsic dynamics (Eriksson 1996; Fischer and Stöcklin 1997). Grassland plants have been shown to persist and occur despite threats such as fragmentation (Maurer et al. 2003).

### 4.1 Species Level Changes That Indicate Homogenisation

In the ISGS, 209 species were recorded across the 12 sites, with 173 being recorded in StableGrass. An 11–16 year period passed (site dependent) between these surveys. The most notable change in species over this time was the loss of 66 species, including specialist species and the gain of 31 species. This, when considered with the statistical analysis, is evidence for increasing homogenisation of the grasslands especially since 10 of the lost species were specialist species (Schrama et al. 2023). Among the species lost was *Coeloglossum viride* (frog orchid), a grassland specialist and classed as near threatened on the Irish Vascular Plant Red List (Wyse Jackson et al. 2016) that has been recorded in less than 100 of Ireland's hectads (10 km × 10 km distribution squares) (Stroh et al. 2020). *Ophioglossum vulgatum* (adder's tongue), a small fern that has only been recorded in 166 hectads, and *Parnassia palustris* (grass‐of‐parnassus), recorded in only 191 hectads in Ireland, were both also absent from StableGrass surveys. Both species are specialists of calcareous wetlands and bordering grasslands. The loss of grassland specialists and species of conservation concern is consistent with studies into changes that have occurred in other European grasslands (Fischer and Stöcklin 1997; Diekmann et al. 2019; Bauer and Albrecht 2020).

Species that have been gained since the ISGS and are indicative of succession: *Pteridium aquilinum* (bracken) was found in the StableGrass survey and not the ISGS. This species is likely to have increased due to more intensive sheep grazing of upland grasslands (Stroh et al. 2020), which is consistent with the field observations in the 2023 StableGrass survey (e.g., Site 6). *Rubus fruticosus agg*. (bramble) was a woody species that was gained since the ISGS. This species is competitive and has responded to a decline in grazing of semi‐natural habitats (Marrs et al. 2010; Stroh et al. 2020), indicating succession in relevés where this species was recorded. *Schedonorus pratensis* (meadow fescue) was recorded in the current survey and is described in the BSBI Plant Atlas as being a species of neutral, usually fertile soils (Stroh et al. 2020). This suggests that the relevés that contained this species may have had a trajectory of increasing fertility. On the other hand, some gains in species could be considered positive for the conservation of the grassland sites. *Dactylorhiza maculata* (heath spotted‐orchid) and *Orchis mascula* (early purple orchid) were recorded during StableGrass surveys but were not recorded in the ISGS. While these are common orchids (644 and 486 hectads respectively (Stroh et al. 2020)), any orchid species is considered a High Quality indicator for the 6410 and 6210 Annex I grassland habitats (O'Neill et al. 2013). Another example of a gained species that would indicate good quality calcareous grassland is *Rosa pimpinellifolia* (burnet rose) (Stroh et al. 2020). This species has been only recorded in 276 hectads in Ireland and is characteristic of sand dune grasslands or limestone grassland (Stroh et al. 2020). While this could be interpreted as woody succession, the cover was less than 25%, which meets the criteria for 6210 Annex calcareous grassland (O'Neill et al. 2013) and may suggest that in this instance, it is not a sign of succession.

### 4.2 Frequency Changes and Homogenisation

Considerable changes in species frequency were observed since the ISGS at both the site and relevé levels. Out of the total species, 111 (46%) declined in frequency between surveys (Table 3). It has been shown in studies that the frequency of specialist species is expected to be higher in old grasslands with minimal management (McCook 1994; Schmid et al. 2017). In line with this, the reduced frequency of these species in the StableGrass survey—while not on its own sufficient to show homogenisation—may provide further supporting evidence of increasing homogenisation. This increase may be due to increased intensity of management, especially in the GS3 sites. For example, at Sites 4, 5 and 6 (upland GS3 sites) overgrazing was observed which may have resulted in low species richness alongside a species composition dominated by graminoid species, consistent with Tóth et al. (2018). Increases in graminoid cover at the expense of specialist forb species is a well‐reported phenomenon in overgrazed grasslands (Grime 1973; Dumont et al. 2011; Varga et al. 2021, 2024; Hempson et al. 2022). For example, in these upland GS3 sites *Galium saxatile* , *Potentilla erecta* and *Euphrasia officinalis agg*. were often the only forb species found in these relevés. This vegetation composition, as a result, is relatively homogenous, demonstrating the increase in homogenisation since the ISGS survey. The dispersion analysis further supports this progression towards homogenisation in the surveyed sites.

It must be noted that factors other than changes in management intensity and homogenisation may have resulted in the changes observed in the species frequency. The climate variability in the intervening period between the ISGS and the StableGrass surveys could have resulted in differences in species composition from year to year (Adler et al. 2006). Dostálek and Frantík (2011) observed increased dominance and richness of graminoid species in dry grasslands after wetter conditions in the winter. Matesanz et al. (2009) observed that long‐term increases and high interannual variability between temperature and precipitation had strong relationships with the trends and interannual variation observed in the plant communities. It must also be noted that only 12 sites, with four of each habitat, that met quite strict criteria within 8 counties of western Ireland were studied, resulting in 44 relevés that were coincident with the 4544 relevés of the ISGS, and therefore the results presented are only a small sample of the total Irish grassland sites.

## 5 Conclusion

While this study identified no significant changes in species diversity over the period 2007–2013 and 2023 in three semi‐natural grassland habitats, an increasing trend towards homogenisation was discovered. This homogenisation is evidenced by an increased overlap in the relevés and habitats in the StableGrass survey when compared to the ISGS. Homogenisation has consequences for the conservation of grassland plant diversity as it reduces the area of quality habitat and number of different niches that different plant species could occupy. There was a 17% loss in the number of species recorded, with some of these species being rarer species that were more sensitive to change. The loss of forbs in combination with an increase in graminoid species indicates a change towards a more homogenous habitat. This increasing trend of homogenisation may over time begin to result in a reduction of the diversity and habitat quality of Ireland's semi‐natural grasslands. There may still be time to reverse this homogenisation through subtle changes in management. Studies have shown that restoration of semi‐natural grasslands to reference conditions is possible but successful restoration diminishes with increasing time (e.g., Stampfli and Zeiter 1999; Joyce 2014). Future re‐surveys of these grassland sites are recommended to better identify trends.

## Author Contributions

**Oliver Lynch Milner:** conceptualization (equal), data curation (equal), formal analysis (equal), investigation (lead), methodology (equal), writing – original draft (lead), writing – review and editing (equal). **Astrid Wingler:** conceptualization (equal), funding acquisition (lead), methodology (equal), project administration (lead), supervision (supporting), writing – original draft (supporting), writing – review and editing (equal). **Fiona Cawkwell:** conceptualization (equal), funding acquisition (supporting), methodology (equal), project administration (supporting), supervision (supporting), writing – original draft (supporting), writing – review and editing (equal). **Karen L. Bacon:** conceptualization (equal), funding acquisition (supporting), methodology (equal), project administration (supporting), supervision (lead), writing – original draft (supporting), writing – review and editing (equal).

## Funding

This work was supported by the Irish Department of Agriculture, Food and the Marine's Competitive Research Funding Programme (2021R529).

## Conflicts of Interest

The authors declare no conflicts of interest.

## Supporting information


**Data S1:** ece373231‐sup‐0001‐Appendix.xlsx.

## Data Availability

Raw data are provided in the Supporting Information.
